# Ethnic markers and the emergence of group-specific norms

**DOI:** 10.1038/s41598-020-79222-0

**Published:** 2020-12-17

**Authors:** Juan Ozaita, Andrea Baronchelli, Angel Sánchez

**Affiliations:** 1 Grupo Interdisciplinar de Sistemas Complejos, Departamento de Matemáticas, Universidad Carlos III de Madrid, 28911 Leganés, Madrid Spain; 2 Unidad Mixta Interdisciplinar de Comportamiento y Complejidad Social (UMICCS) UC3M-UV-UZ, 28911 Leganés, Madrid Spain; 3 Department of Mathematics, City, University of London, London, EC1V 0HB UK; 4 The Alan Turing Institute, British Library, 96 Euston Road, London, NW12DB UK; 5 Institute UC3M-Santander for Big Data (IBiDat), Universidad Carlos III de Madrid, 28903 Getafe, Madrid Spain; 6 Instituto de Biocomputación y Física de Sistemas Complejos (BIFI), Universidad de Zaragoza, 50018 Zaragoza, Spain

**Keywords:** Nonlinear phenomena, Computational science

## Abstract

Observable social traits determine how we interact meaningfully in society even in our globalized world. While a popular hypothesis states that observable traits may help promote cooperation, the alternative explanation that they facilitate coordination has gained ground in recent years. Here we explore this possibility and present a model that investigates the role of ethnic markers in coordination games. In particular, we aim to test the role of reinforcement learning as the microscopic mechanism used by the agents to update their strategies in the game. For a wide range of parameters, we observe the emergence of a collective equilibrium in which markers play an assorting role. However, if individuals are too conformist or too greedy, markers fail to shape social interactions. These results extend and complement previous work focused on agent imitation and show that reinforcement learning is a good candidate to explain many instances where ethnic markers influence coordination.

## Introduction

Despite the globalized nature of our societies, ethnic groups behaving according to disparate social norms or conventions are ubiquitous^1^. According to Barth^2^, people identify themselves, and are identified by others, as belonging to certain ethnic group by means of culturally transmitted features, such as wealth symbols, language, culture and artistic forms, dress style or cuisine. All these attributes have in common that they are external and can be seen, evaluated and acted upon by any other member of the population. In turn, mechanisms based on social categorization and parochialism work to maintain them^3^.

Several researchers argued that ethnic groups are the basic locii for cooperation, enabling individuals to profit from cooperative exchanges depending on the observation of the traits displayed by others^4–6^. However, the existence of cooperative or altruistic disposition towards similarly marked individuals has been challenged based on the possibility of free-riding^7^. An alternative explanation solves the free-riding problem by maintaining that the social role of ethnic markers can be to facilitate coordination rather than cooperation^8–10^. While there is contradictory experimental evidence on this hypothesis^11–14^, research specifically designed to test between these two options seems to favor the coordination interpretation^15^.

In this paper, we focus on the role of ethnic markers on norm formation, modelled as a game of coordination^18–24^. Our aim is to study reinforcement learning^25–27^ in this context, motivated by recent empirical findings that have identified this evolutionary mechanism in many instances of human interaction^28,29^. We work in the framework introduced by McElreath et al.^9^ consisting of a unique binary marker used by individuals to choose their behavior in social interactions . This characterization of strangers based on ethnic markers is thus used to determine whether or not there are shared social norms with them^16,17^. Importantly, their model was based on imitation-driven evolutionary dynamics, in which populations using a given strategy (marker-dependent or not) grow or decrease depending on the difference between their payoff and those of the other populations. In contrast, our approach based on reinforcement learning does not use the information on payoffs from other individuals in the population, which is more often than not unavailable, and is governed only by the individual’s own aspirations and payoff.

Indeed, the rationale for exploring reinforcement learning as an alternative to proportional imitation is that, while the first has been criticised for assuming excessive flux of information in the population, the latter allows to achieve a cooperative equilibrium by using only individual information^30–32^. In our model, each agent is defined by an individual parameter called the aspiration. This parameter defines how the outcome of an interaction makes it more likely to choose a given action in the future, when the payoff it yields is larger than the aspiration (positive stimulus). The opposite occurs when the payoff is smaller thant the aspiration (negative stimulus), namely the action becomes less likely to be repeated. In the following sections, we explore the role of this parameter, as well as other parameters arising from the model introduced in Ref.^1^. We will thus identify the conditions under which markers promote coordination, which will be summarized in the final discussion.

## Model

In this section we introduce our model for the marker’s mechanism incorporating reinforcement learning^25–27^ as our paradigm for the evolution of the agents decisions. As stated above, the key feature of this dynamics is the aspiration parameter, which agents use to evaluate the outcome of a round. If the outcome is above (below) their aspirations, they generate a positive (negative) stimulus and tend to repeat (avoid) their previous action. The effect of this dynamics was first studied by Macy and Flache in Ref.^26^ where they considered two-agent interactions through different social dilemmas, one of them being coordination. Without considering markers, they showed that aspirations that can generate positive and negative stimuli tend to promote cooperation in social dilemmas; in the case of coordination, this amounts to largely increasing the proportion of interactions in which agents succeed in coordinating. Other choices for the aspiration values led in turn to coordination failures. Here, we consider different aspiration values when markers mediate a coordination game played by a population of size $N>>2$. When markers are present, individual actions evolve based on two different inputs, namely from intra- and iter- marker interaction, which significantly increases the complexity of the possible outcomes.

In our model, agents are characterized by the following parameters:

- *Behavior* It is the action chosen for the coordination game. It may take two different values $\left\{ 0,1\right\}$.
- *Marker* It is a visible characteristic which identify the interaction. It may take two different values $\left\{ 0,1\right\}$. In our version markers will be immutable, modeling observable social traits that either do not change or change in a very slow timescale.
- *Aspiration* The payoff expected by the agent, which will define the stimulus that it receives from the interaction.
- *Probability vector* The probability for an agent to choose a behavior according to the subject’s marker. We consider the probabilities to choose an action when interacting with an agent with the same marker and the same for the case of an agent with the opposite marker, i.e.,
  $$\begin{aligned} p_{=,0}+p_{=,1}= & {} 1, \\ p_{\ne ,0}+p_{\ne ,1}= & {} 1. \end{aligned}$$

In this model, population is split in couples and agents interact via a coordination game. These games are characterized by a symmetric payoff matrix, such as

1 $$\begin{aligned} \begin{pmatrix} 1+\delta &{}\quad 1 \\ 1 &{}\quad 1+\delta \end{pmatrix}, \end{aligned}$$

where each element of the matrix ($a_{ij}$) corresponds to the payoff obtained by player 1 when choosing behavior *i* while player 2 chooses strategy *j*. When both players choose the same action ($a_{ii}$ for $i=1,2$ is selected) they get a better payoff given by the additional amount $\delta$. We then say that they have coordinated in that timestep. Note that markers do not affect the payoff matrix; their effects are only included in the probability vector that we described above.

On the other hand, we have included in the model several variables, mostly taken from the pioneering research about markers in Ref.^1^, that characterize the population and their interactions as a whole:

- *e*: Probability for the interaction to be marked, modelling the willingness of the agents to choose someone with the same marker. This parameter takes into account the tendency to bias interactions towards the marker we share with other members from our population, or in other words, the degree of homophily^33^.
- $\delta$: Extra payoff for successful coordination. In the following sections, we will set $\delta =0.5$.

The model consists of *N* agents , that evolve in time according to the following dynamics:

1. *Select type of interaction* With probability *e*, the individual interacts with another one chosen at random without making any reference to the marker, and with probability $1-e$ the individual interacts with another one who shares her same marker. This implies that interactions with others sharing one’s marker take place with probability $1-e/2$. Once the type of interaction is selected, a random couple that fulfill the chosen criterion is assigned to the focal individual.
2. *Select individual actions and play the game* Both interacting agents select a behavior according to their probability vector and play the coordination game accordingly.
3. *Collect payoff and update probability vector* Both agents collect their payoff. If this payoff satisfies their expectations, they generate a positive stimulus, and vice-versa. A positive stimulus encourages the agent to repeat the same action and a negative one repel him. These dynamics are captured in the following equations, extracted from Ref.^26^:
  2 $$\begin{aligned} p_{a,t+1}= \left\{ \begin{array}{lcc} p_{a,t}+(1-p_{a,t})ls_{a,t} &{}\quad {\mathrm{if}} &{} s_{a,t}\ge 0, \\ \\ p_{a,t}+p_{a,t}ls_{a,t} &{} \quad {\mathrm{if}} &{} s_{a,t}<0, \\ \end{array} \right. \end{aligned}$$
  where $p_{a,t}$ is the probability of choosing a certain action at the timestep *t* and $s_{a,t}$ is the stimulus obtained by the agent at that timestep. Furthermore, *l* is the learning rate^26^, that controls the speed of adaptation of the agents.

Before going into the presentation of our findings, a brief discussion is needed to understand the methodology of the present work. As can be seen from the above description, we have introduced a model that incorporates the mechanism we want to understand, but at the same time it involves a number of parameters and variables. This immediately raises two questions: first, how robust are the conclusions we can draw from the model with respect to changes in the parameters, and second, what is the range in which those conclusions hold. To answer these questions, we must resort to a detailed exploration of the model, checking what happens with its different alternatives (such as variations on the perfection of the coordination game, variations on the learning rate, variations on the bias of the interaction, heterogeneous aspirations, and others we discuss below). While at first glance this may look like a collection of simulations, we have made every effort to address all possibilities and provide a complete study of our model. As will be shown below, this thorough simulational study will allow us to establish our conclusions on firm grounds.

## Results

We present here our main results for the model above. First of all, we will consider that all agents have identical aspirations and we will characterize the different collective behaviors arising. Once the baseline model is well understood, according to our program we will study variations on the parameters . We will also analyze the effect of modifying the payoff matrix (imperfect coordination), learning rate variations (*l*), assortment effects (more than one aspiration present in the population), migration effects and variations on the bias of the interaction (*e*). Unless otherwise specified, the values of the main parameters and variables of the model are as follows: Learning rate $l=0,5$ (fast learning), $e=0.5$ (interaction is biased towards individuals with the same marker, and a unique population of $N=500$ agents. We run simulations for 4000 interactions per agent (in total, $3\times 10^{6}$), and statistical averages and plots have been made with 100 simulations with markers and behaviors initially chosen at random with equal probability.

**Figure 1:**
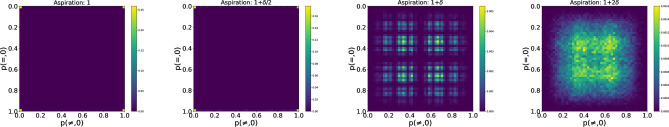
2D histograms for the probability vectors of the population. Shown is the fraction of agents with specific values of choosing action 0 when facing another agent with different marker (horizontal axis) and when facing another agent with the same agent (vertical axis). From left to right, aspiration levels are 1, $1+\delta /2$, $1+\delta$, $1+2\delta$.

### Baseline

We start by looking at the evolution of the probability vector phase space for different values of the aspirations. Specifically, we consider the cases $A_{i}=\left\{ 1, 1+\delta /2, 1+\delta , 1+2\delta \right\}$, which represent aspiration levels at the minimum payoff(and therefore always satisfied or neutral), an intermediate value between payoffs, the maximum payoff, and a level above all payoffs (which leads to negative stimulus for all outcomes), respectively. Results are shown in Fig. 1.

We can distinguish two main kinds of behavior depending on the value of aspiration: For $A_{i}\le 1+\delta /2$, the system is dominated by positive stimuli. Reinforcement learning leads agents to deterministic behavior, sticking to one of the actions for each of the two possible interactions, individuals with the same marker and with different markers. On the contrary, when $A_{i}>1+\delta /2$, the learning process is dominated by negative stimuli, and agents behave more randomly, meaning for both categories agents may choose one or other strategy with nonzero probability.

In order to shed further light into the different regimes, we introduce the *ratio of coordination* $m_{i}$ defined as

3 $$\begin{aligned} m_{i}=\dfrac{\text{ no. of coordinations }}{\text{ no. } \;\text{ of } \;\text{ interactions }} \end{aligned}$$

**Figure 2:**
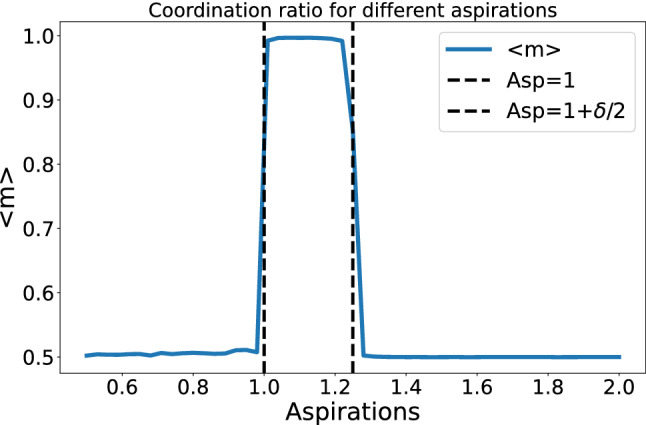
Coordination ratio $m_{i}$ for different values of the aspiration, averaged over agents, time and simulations.

The ratio of coordination allows us to monitor how successful the average player is in coordinating with every individual she meets. In Fig. 2 we show the values of this parameter averaged over the length of the run and over simulations for an interval of values for the aspiration level. This figure allows us to clearly identify the separations between the three regimes we have identified:

The first regime arises when $A_{i}\le 1$: All stimuli are positive (or neutral, in the extreme case). This means that every action encourages the subject to repeat the same action. If we let this dynamics evolve, the subjects end up organized in the four possible combinations of $\left\{ p_{=,0},p_{\ne ,0} \right\}$. Note that the agents always receive the same positive stimuli irrespective of their behavior, resulting in no correlation strategy-marker. We will refer to this type of behavior as *frequentists* referring to the fact that their choices depend on the agent and its sequence of interactions. As a consequence, the ratio of coordination is around 0.5, as expected in such a random setting. The second regime arises when we increase the aspiration further, $1<A_{i}<1+\delta /2$. Here, both positive and negative stimuli exist but the former dominate. Agents are again clustered, but this time $p_{=}$ is marker related and $p_{\ne }$ is unique for all the population. If both behaviors are the same, homogeneity is promoted. At the end of the simulations, we observe that agents develop intra-marker correlations (collective organized strategy within each marker subgroup) and inter-marker correlations (collective organized strategy for interactions between marked subgroups). Thus, agents have a criterion for both categories.

**Figure 3:**
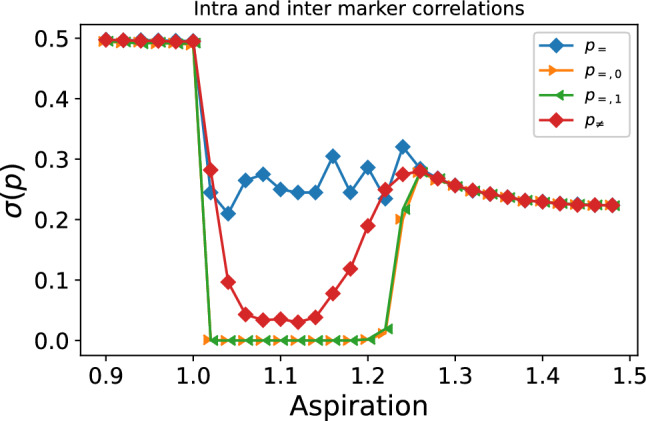
Standard deviation of $p_{=},p_{\ne },p_{=,0}$,$p_{=,1}$averaged over agents and simulations for the relevant range of aspirations. The parameters used are the same of Fig. 1.

Further information on the correlations between markers and behavior can be obtained from the study of the variance of the agents probability vector for the relevant range of aspirations in the stationary state. In order to show the intra-marker correlation, we plot the averaged (over agents and simulations) standard deviation of $p_{=},p_{\ne }$. To show the marker correlation, we plot the standard deviation of the probabilities arising in the population with marker 0 ($p_{=,0}$) and marker 1 ($p_{=,1}$). As we can see, in the regime $1<A_{i}<1.25$ the standard deviation for both markers drops independently to zero. This means that the stationary distribution of $p_{=,0}$ and $p_{=,1}$ tends to a unique value, which, as seen in Fig. 1, it is either zero or one. On the other hand, as the standard deviation of $p_{=}$ is nonzero, this concentration around a single value occurs independently for each marker, capturing the marker correlation. The inter-marker correlation also achieves low variances, but never reaching zero, which shows the weakness of the correlation, arising from the parameter *e* and the bias in the interaction. Still, agents in this regime present lower standard deviations than the ones with higher and lower aspirations, showing us that they are correlating the marker of their partner with a particular action. We will call the agents in this regime *learning* agents, as they do learn to coordinate; note that the ratio of coordination is almost 1 (cf. Fig. 2), the difference being due to mistakes or, equivalently, fluctuations during the learning process (Fig. 3).

Finally, the third regime occurs when $1+\delta /2\ge A_{i}\le 1+\delta$. We enter the stochastic behavior: Positive and negative stimuli exist, but negative ones dominate. Probability vectors are uniformly distributed in the phase space. Agents do not share a criterion nor have a clear strategy for any of the categories. We will call them *random walkers*. The ratio of coordination is again 0.5, as in the case of frequentists.

### Variations on the baseline

Having described in detail the phenomenology we observe on our baseline model, in this subsection we are going to study the role of the parameters we of the model by varying them one by one and analyzing the change in the qualitative results.

#### Variations on the perfection of the coordination game

In the baseline, both actions lead to the same payoffs when the agents agree on them. However, it is possible that the two choices lead to different payoffs, i.e., the interaction is not pure coordination anymore. We explore this scenario by modifying the payoff matrix as follows.

4 $$\begin{aligned} \begin{pmatrix} 1+\delta +a_{1} &{}\quad 1-b_{1} \\ 1 &{}\quad 1+\delta \end{pmatrix} \end{aligned}$$

**Figure 4:**
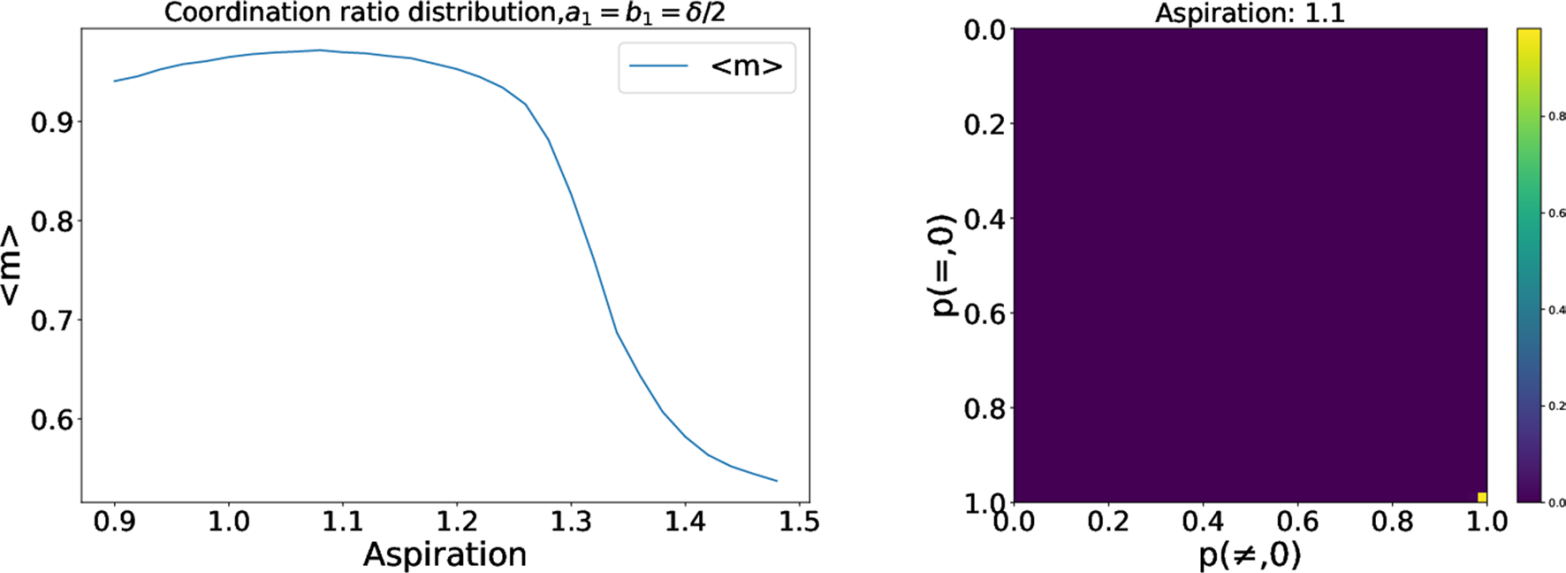
Evolution of the average ratio of coordination for the asymmetric coordination problem with $a_{1}=b_{1}=\delta /2$ (left) for different aspiration levels, and 2D histograms for the probability vectors of the population (right) for $A_i=1.1$.

We simulated, as an example, $\delta =0.5, A_{i}=1+\delta /2, a_{1}=b_{1}=\delta /2$, obtaining the results shown in Fig. 4. Agents choose the Pareto-dominant equilibrium (the one with the largest payoff) if their aspiration level is not too large: Reinforcement learning agents organize around this strategy, maximizing their individual and global payoff. The 2D histogram shows clearly that all agents choose the best equilibrium irrespective of the markers. Therefore, markers play no role when different outcomes have different payoffs because higher payoffs provide higher stimuli, which is the key element for an agent in order to arrive to the final equilibrium. Thus, markers are only relevant to self-organize the system when there are several equilibria with the same payoff in a pure coordination game, helping agents organize in categories $\left\{ 0,=,\right\}$, $\left\{ 0,\ne ,\right\}$, $\left\{ 1,=,\right\}$,$\left\{ 1,\ne ,\right\}$, which is not possible in the absence of markers. Categories arising from markers define the new collective agreements or conventions that are present in the population. It has to be kept in mind that in addition to the two equilibria being equally beneficial, agents must have the right aspiration value, for positive stimuli to lead to the evolution of coordinated behavior.

**Figure 5:**
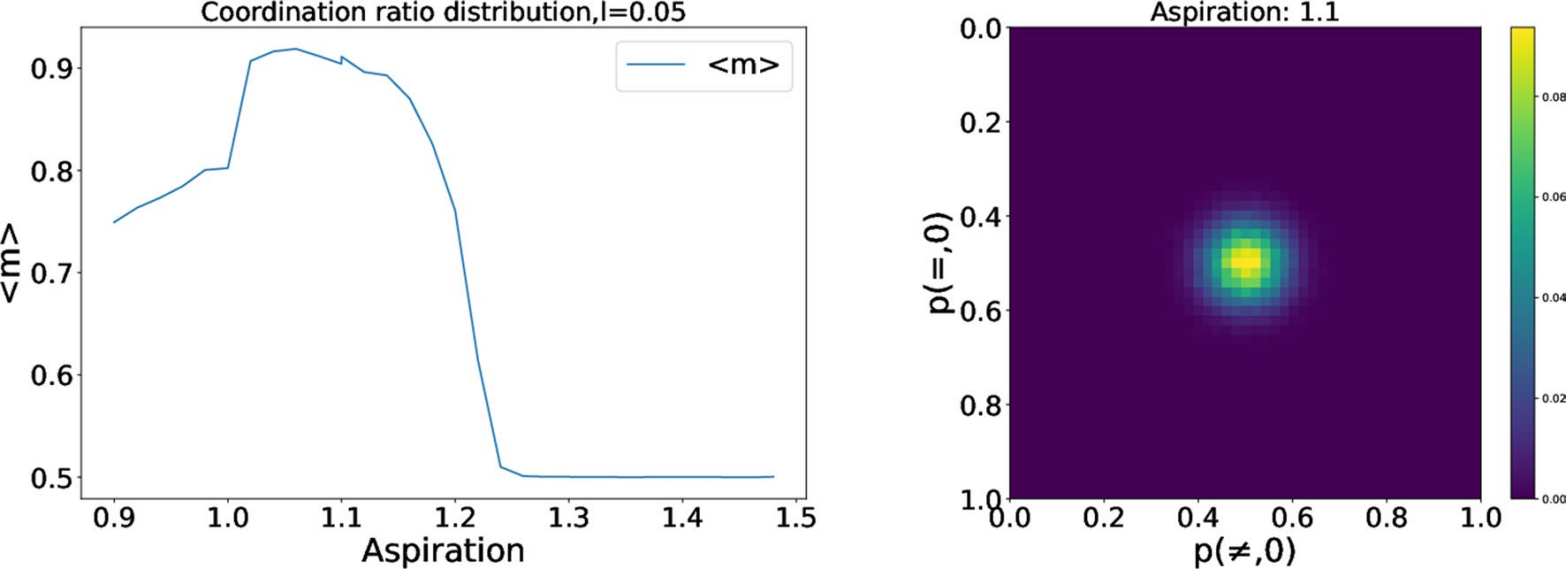
Left: Coordination ratio $m_{i}$ for different values of the aspiration, averaged over agents, time and simulations. Right: 2D histograms for the probability vectors of the population. $l=0.05$.

#### Variations on the learning rate (*l*)

In the baseline simulations, we have used fast learning, i.e., agents react strongly to the stimulus. Let us now look at the possibility that qualitative results, like the marker correlations, are the same if the learning rate is slower ($l=0.05$). The results are summarized in Fig. 5. The main difference with the previous case is the higher level of coordination among the populations with low aspirations. With a low learning rate, the influence of stimuli is very weak. Agents need more interactions to achieve a stationary probability vector, which is expected for the equilibrium in agents with low aspirations. This increase in the number of interactions promotes a more homogeneous equilibrium. It is interesting to note that if we could take the limit $l\rightarrow 0$ our agents would take infinite time to arrive to an equilibrium, but it would be entirely homogeneous. We can conclude then than, at least in the variable $m_{i}$, the qualitative difference in behavior around $A_{i}=1$ can become continuous if we choose the proper learning rate. The variation on the learning rate can be also noted in the high aspirations region. As the learning rates are smaller, random-walkers stay trapped around quasi-random behavior, with probabilities close to 0.5.

**Figure 6:**
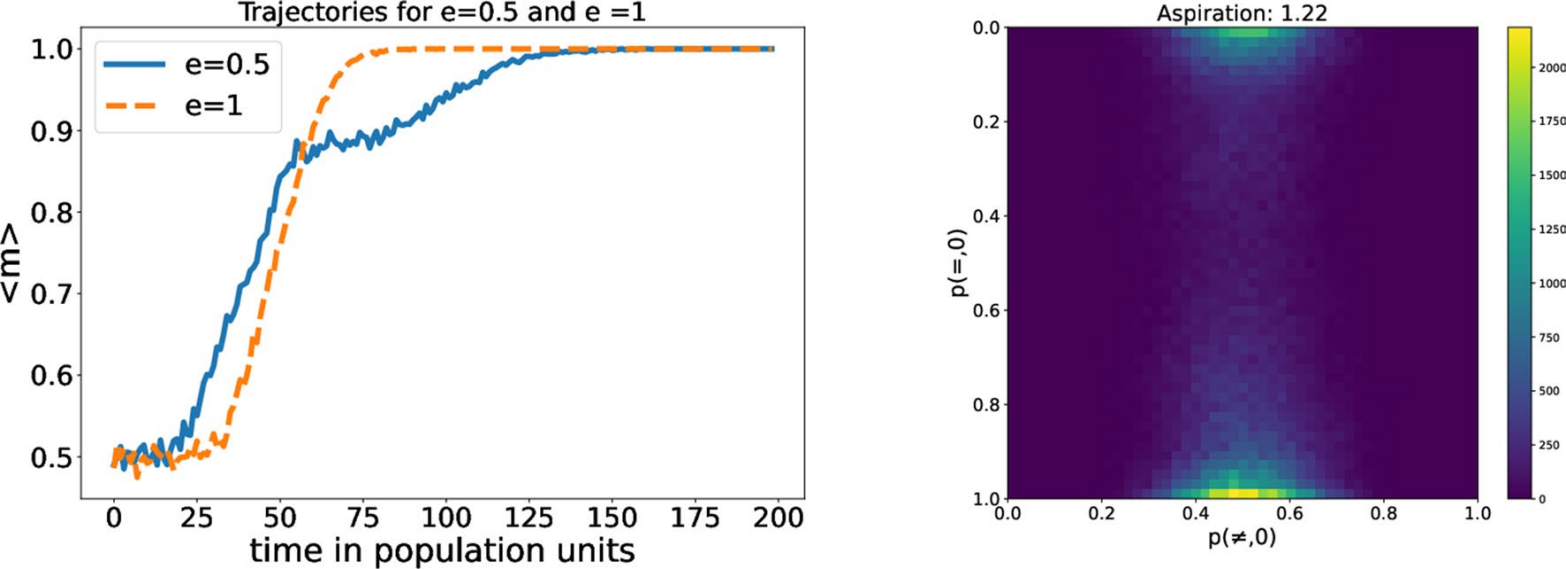
Left: Average coordination ratio for $A_{i}=1.1$ and $e=0,5$ or $e=1$ . Right: 2D histograms for the probability vectors of the population. $A_{i}=1,22$

#### Variations on the bias of the interaction (*e*)

Another important feature of our model is the bias on the interaction. In order to discuss the effects of this, we consider an example of the evolution of the coordination rate in the population. We choose a population with a global aspiration $A_{i}=1,1$ to promote coordination, and check, for a slow learning rate ($l=0,05$), the difference between $e=0,5$ and $e=1$. In Fig. 6, we can see that there are two different coordination velocities, represented by the different slopes of the curve. There are two stages in the process of arriving to the equilibrium: In the first one, the system is not organized and it is building both criteria, the intra-marker one and the inter-marker. In the second one, the intra-marker criterion exists, but the agents do not know yet what to do when they interact with someone with a different marker. The bias can also be seen in the phase space of probability vectors . If we study the statistics of the previous case, for comparison, we can see that the biased case has some kind of transition in two stages, from full coordination to random walkers. Firstly, the inter-correlation is broken so the agents spread around one of the axes. In a second stage, the intra-correlation is destroyed and we arrive to the homogeneously distributed phase space. On the other side, if there is no bias, both correlations are destroyed at the same time.

**Figure 7:**
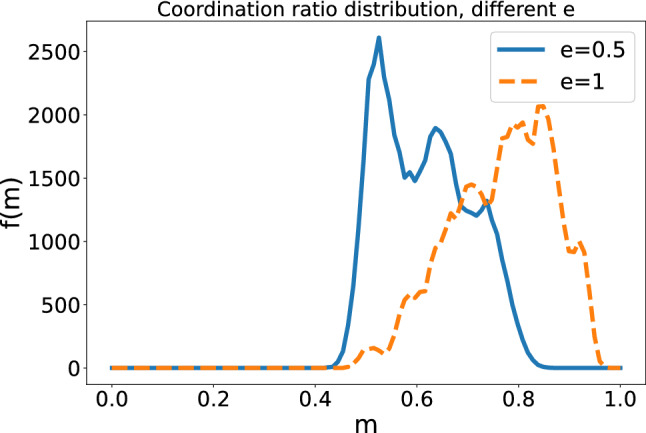
Histograms of the coordination ratio for $A_{i}=1.22$ and $e=0,5$ or $e=1$.

Further information can be obtained from the coordination picture near the transition between learning agent and random walker, shown in Fig. 7. As it could be seen also in the probability vector phase space, in the biased case the weak correlation is destroyed and the stronger one stays. In the unbiased case, both transitions persist at the edge of the transition. And, as we can see, the coordination distribution has a positive skew in the biased case and a negative one in the unbiased. We can say then that the strength of the correlation is interaction based, as weakening one of the correlations makes for a higher probability of obtaining lower levels of coordination.

#### Heterogeneous aspirations

In order to study heterogeneous, diverse populations we are going to choose three different values for the aspirations, representing the three main categories we have analyzed above. These values will be $A_{i}=\left\{ 0.8,1.1,1.5 \right\}$ and they will appear in the population with different proportions. We will study populations with two different categories of aspirations in different proportions ($25\%,50\%,75\%$) and populations with three different categories of aspirations in an equal proportion. These configurations will be studied under baseline conditions of fast learning rate ($l=0.5$) and marked-biased interaction($e=0.5$), with all the simulation parameters as explained in the former section.

**Figure 8:**
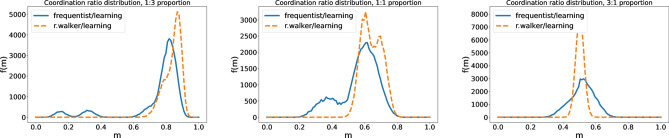
Coordination ratio for a a frequentist/learning or a random walker/learning agent population, with ratios (1:3) (left), (1:1) (middle), and (3:1) (right).

In general, our results show that when learning agents are mixed with other type of agents they lose the full coordination they achieve when they are on their own, as described in the case of the baseline simulations. However, there are other consequences of this mixed population that change depending on the precise composition considered. Thus, if learning agents are a majority (75% of the population), intra and inter-marker correlation still exist but they are not perfect. This means that $0.5<p{=,i}<1$, $0.5<p{\ne ,i}<1$, so their most probable option is to choose the correlated probability, but they could break the rule (leading to only partial correlation). In turn, the global level of coordination depends on who are they mixed with: If they are mixed with frequentists, Fig. 8 shows that they self-organize in the corners of the probability phase space, i.e., on deterministic behavior, the specific one arising depending on their history of coordinations. It is interesting to note that four groups of frequentists are formed, depending on their coordination with the collective agreements of the learning agents. The widest peak corresponds to the one with the maximum coordination, probably because of the influence of the learning agents. On the other hand, if they are mixed with random walkers, these last ones eventually cluster in the surroundings (in the probability vector phase space) of the collective agreement made by the learning agents. In addition to the random movement from the interactions between them, they are driven by the learning agents to their positions, as they prefer coordination to uncoordination.

Now, in case learning agents are in 1:1 proportion, what we observe in the center panel of Fig. 8 is that the inter-marker correlation is destroyed, while the intra-marker still survives, but partially affected too. The coordination ratio is lower than before, but higher than the random value of 0.5. If they are mixed with frequentists, they self-organize with an individual criterion that is affected by the presence of learning agents (just like the previous case). The absence of the inter-marker correlation can be noticed in the fact that the coordination ratio for the frequentist population is a sum of two Gaussian distributions, one related to the ones that share the learning agents intra-marker correlations and the ones that do not. If, on the contrary, they are mixed with random walkers, they stay at the surroundings of the learning agents criterion, as before. However, as the inter-marker correlation has been destroyed, the surroundings are a whole dimension from the probability vector phase space. The coordination ratio is still higher than 0.5 even for random walkers.

Figure 8 shows in its right panel results for the situation in which learning agents are a minority ($25\%$). In this case, both of their correlations are very weak or suppressed. If they are mixed with random walkers, both correlations are destroyed. However, if the majority are frequentists, learning agents stay in the surroundings of the equilibria that frequentists have decided, achieving a slightly better coordination ratio. This occurs because they coordinate with a group of frequentists and have a nonzero probability of coordinating with learning agents and frequentist groups.

Finally, in a well mixed population of agents with fixed aspirations and three choices, namely $\left\{ 0.8,1.1,1.5 \right\}$, the results are similar to the case in which learning agents were in the minority. This means that as before, they tend to stay in the surroundings of the groups created by frequentists.

#### Variations on the number of groups and role of migration

An important question as to the effect and dynamics of behaviors in marked populations, already raised in Ref.^1^, is the possibility that there are different populations, possibly separated geographically, a situation that may lead to several combinations of markers and actions, not necessarily agreeing between groups of individuals. Therefore, we are now going to consider this issue, and to that end we will consider that agents have an individual label indicating to which group they belong to. Following Ref.^1^, we introduce two migration parameters, *m* and $\beta$, representing the proportion of population that migrates in each group (*m*) and the frequency of the process($\beta$), respectively. Specifically, migration events take place every $\beta N$ rounds at the end of the corresponding timestep, and in each migration event *m* individuals in each group are randomly chosen and also randomly reassigned to other groups keeping the population of each group constant.

As the simplest way to understand the effects of migration, we will consider two separate population groups, denoted by a binary label $\left\{ 0,1 \right\}$. We have considered medium-sized populations ($N_{group}=250$), while all the other parameters have been set like in baseline case. In what follows we study two cases: First, two groups with different initial aspirations, so we can assess the role of the spatial structure in the conformation of marked communities, and a second situation with the same fixed initial aspiration for both groups, studying how the amount and velocity of migration influence reaching or not homogeneity between groups in the collective agreements.

**Figure 9:**
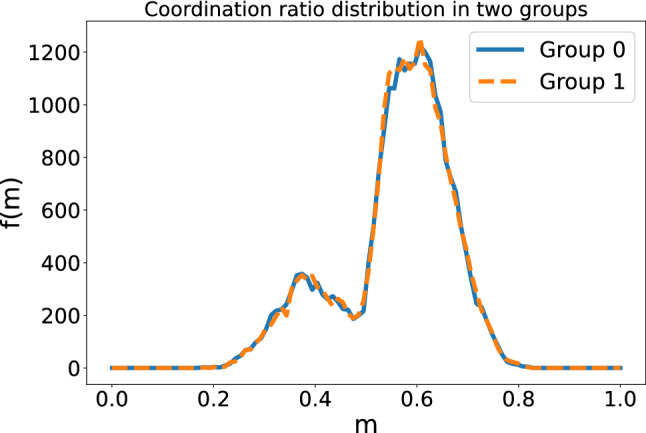
Coordination ratio for group 0 and for group 1.

For the first study, we have in turn considered two possibilities, one with group 1 with initial aspiration 0.8 and group 2 with initial aspiration 1.1, and another one with group 1 with initial aspiration 1.5 and group 2 with initial aspiration 1.1. Our results are summarized in Fig. 9, where it can be seen that the existence of separate groups, which as we said can come from the underlying spatial structure, does not promote new results nor marked communities as, at the end of the day, we obtained two fragmented groups whose main features are basically the same as the ones studied above.

For the second study, namely the influence of the migration parameters, we set up two groups of learning agents with fixed aspirations ($A_{i}=1.1$) with different migration speeds ($\beta$) and a fixed quantity of migrants. To be specific, we will use a proportion of population that migrates in each group given by $m=1/N$. With this setup, we have explored the number of coincidences in the possible criteria for the intra-marker and inter-marker correlations. We define an intra-marker coincidence as the use of the same behaviors between groups in interactions between agents of the same marker, and inter-marker coincidence as the use of the same behaviors between groups in interactions between agents of different markers. Therefore, we define the intra/inter-homogeneity as the percentage of realizations for a configuration that promotes these coincidences.

It has to be taken into account that, as we are using $e=0.5$, a $75\%$ of all interactions are intra-marker so it is very likely that intra-marker correlations will evolve on a faster time scale. On the other hand, in principle one should expect that migration leads to more heterogeneity and to longer time scales for the arising of conventions, because agents migrate between groups having possibly different actions for the same marker. However, our simulations show that this is not the case and migration turns out to be compatible with homogeneity and a single time scale, which means that the outcome of the evolution is not affected by the bias towards interacting with one’s own marker. The difference between the numbers for intra and inter homogeneity arises from the fact that the inter-marker correlation is collective agreement in a group, while the intra-marker correlation exists for every marked group (it is proportional to the number of groups). As can be seen in Table 1, the ratio 2:1 for these quantities remains more or less constant until the parameters allow to reach global homogeneity.

**Table 1:** Number of coincidences in collective agreements between different groups for different values of migration speed ($\beta$).

$\beta$	$\%$intra-homogeneity	$\%$inter-homogeneity
No migration	20	45
10	21	48
1	28	60
0.1	50	100
0.01	100	100

In the former setup we were working with $m=1/N$, just because it is the scenario where differences can arise easily before reaching homogeneity. As expected, if we introduce larger fractions of migrants (larger *m*), higher homogeneity arises. For instance, when $\beta =0.1$, for different *m* we obtain the results collected in Table 2, showing the efficiency of migration to induce homogeneity across groups.

**Table 2:** Number of coincidences in collective agreements between different groups for different values of migrating fraction of population (*m*).

*m*	$\%$Intra-homogeneity	$\%$Inter-homogeneity
No migration	20	45
1/*N*	50	100
2/*N*	85	100
5/*N*	100	100
10/*N*	100	100

## Discussion

In this work, we investigated reinforcement learning in a model for the emergence of marker-related behaviour in coordination problems, showing that it can provide a mechanism in order to solve such multi-agent coordination games. We found that

- Markers allow to resolve the (pure) coordination problem through reinforcement learning.
- For coordination to take place, however, aspiration levels must be in the mid range, i.e., not too low and not too high (see also^1^).
- When equilibria are not equivalent, markers become irrelevant.

Furthermore, (1) we found that migration and spatial structure do not promote new collective effects in this context; (2) we checked that results are mostly insensitive to the group size, and that they hold for evolutionary-relevant small groups (see “Appendix”); (3) we also checked that when there is habituation^26^, allowing aspiration levels to change as a function of the received payoffs, a large fraction of agents end up with aspirations beyond the range where learning takes place and random behavior arises, thus breaking the collective behavior related to markers (see “Appendix”).

It is interesting to consider our work in relation to the pioneering proposal by McElreath et al.^9^, which did not consider reinforcement learning but rather imitation-driven dynamics. Our results are largely different from theirs: Indeed, in our model marker-behavior correlations arise less often, while the existence of more than one group or a spatial structure is less significant. The reason for this is that we are considering a dynamics for the actions and, importantly, markers do not evolve in our model. Reinforcement learning dynamics with well tuned aspirations introduces a systematic way for agents to create correlations with markers, while in Ref.^1^ this happens to be an equilibria between different processes of the dynamics. The interpretation of this dynamics is also important: Copying the fittest individual’s action (and possibly marker) may not be a realistic circumstance, as some social features can not be changed by adaptation, or even if they can, individuals may not have information about the payoff obtained by every other individual. Reinforcement learning dynamics only uses individual information, avoiding these methodological issues.

Markers have also been considered in the literature about tag games and social norms in agent-based modelling. Axtell, Epstein and Young^35^ studied tags as promoters of social norms, intended as self-enforcing patterns of behavior. In the framework of bargaining, pairs of individuals play a Nash demand game with three options. Agents update their memories: They remember a number of past interactions and form expectations based on the frequency with which they met each demand. When there are two tags for the population that can also be remembered and associated to different demands, Axtell et al. obtain similar results to ours for pure coordination, in the sense that different behaviors for inter- and intra-group interactions appear, connected to lower or higher payoffs. When coordinating on the two actions yields different payoffs they still observe marker dependent behavior while we do not, which points to evolution being linked to memory in their model as the reason for the contrasting results. Moving further away from our basic setup, the idea of tags as cooperation facilitators has been studied extensively. Edmonds and Hales^36,37^ defined tags that take continuous values. Individuals can produce resources of one kind but they need all the types produced in the population to survive. Continuous tags are used to identify individuals to share resources with. Agent-based simulations with evolutionary dynamics show that in this setup cooperation is not viable over the long run without some new individuals entering the population from outside, although groups of sharing individuals do persist in the medium term. Comparison with our model raises the question as to what would be the fate of coordination with a similar scenario of continuous tags.

Therefore, it is clear that after the pioneering works we have discussed in the early 2000s, there is a wide field to explore about the role played by markers in the emergence of group related behavior, particularly of coordination, and the corresponding factors influencing it. Thus, our research paves the way to study new game structures that may represent new social processes, or to new marker structures that represent more complex social definitions than the binary one we have used (several features may be added: Global markers, memory *à la* Axtell et al.^35^ continuous ones such as those in Refs.^1^). Even markers evolving also by reinforcement learning could be incorporated to our setup to study situations in which such change is actually easy in the society. External observable markers are, according to our model, features that may affect the way a society works. Agreements are reached inside different groups in the society, and this may in turn lead to a more complex understanding of collective social norms.

## Figures and Tables

**Figure 10:**
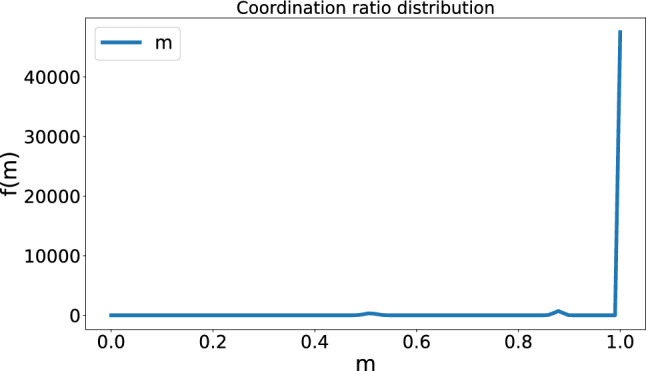
Coordination ratio (left) and aspiration (right) distributions. $A_{0}=0.8$ and $h=10^{-3}$.

**Figure 11:**
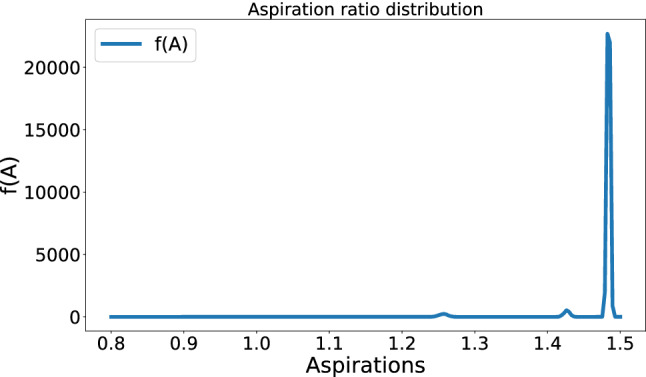
Coordination ratio (left) and aspiration (right) distributions. $A_{0}=0.8$ and $h=5\times 10^{-2}$.

**Figure 12:**
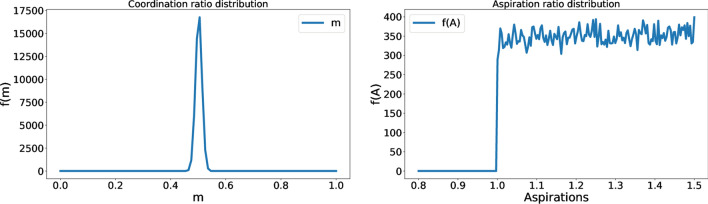
Coordination ratio (left) and aspiration (right) distributions. $A_{0}=0.8$ and $h=0.5$.

**Figure 13:**
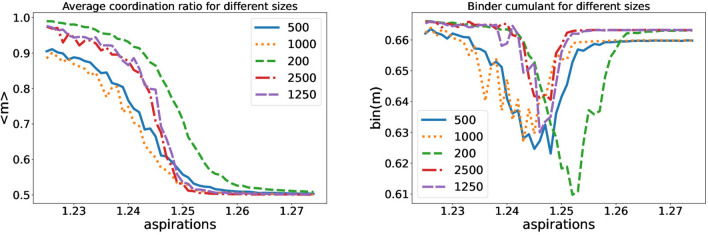
Average coordination ratio (left) and Binder cumulant (right) for several sizes in the $1+\delta /2$ region.

## Appendix

### Reinforcement learning with habituation

For the sake of completeness, it is interesting to explore the effect of dynamic aspirations. It is quite common that individuals have some aspiration level at the beginning of some interaction process, and that as the interactions proceed they get used to some level of payoff and change their aspirations accordingly. This is generally referred to as habituation, this is, the adaptation of the aspiration to the average payoff obtained by the agent. This is introduced by adding a dynamical rule describing the evolution of the aspiration, given by^26^:

5 $$\begin{aligned} A_{t+1}=(1-h)A_{t}+h\pi _{t}, \end{aligned}$$

where $\pi _{t}$ is the payoff obtained by the agent at time *t*. The process of updating individual aspirations is introduced in the dynamics for all the timesteps. After a couple of individuals has interacted and collected their payoff, they adapt their aspirations according to the former equation.

In order to understand the possible new effects introduced by this dynamic adaptation of agents, we will consider the case of one initial aspiration for all the population, with values $\left\{ 0.8,1.1,1.5 \right\}$ as before, and also two different aspirations in the initial conditions in different proportions. We will use a fast learning approach ($l=0.5$) and marked-biased interaction ($e=0.5$) in a single group. Our main findings can be summarized as follows.

When aspirations are initially lower than $1+\delta /2$, and habituation is low (in our case $h<5 \times 10^{-3}$), the dynamics modifies slightly the level of aspirations, but it does not change the qualitative results. Correlations exist and they induce a high level of coordination, which ends promoting high aspirations (agents reach $A_{eq}\simeq 1+\delta$, cf. Fig. 10).

Moving now to intermediate values of habituation ($5 \times 10^{-3}<h<0.5$), we observe in Fig. 11 that habituation destroys correlations, diminishing the coordination ratio and eventually taking the system to uncoordination. As we are biasing the interaction, there is a two-stage destruction. This process happens stochastically, there is not a critical value of *h* that separates both behaviors. Finally, for high values of *h* (in our case $h\sim 0.5$), it can be seen in Fig. 12 the system tends to a uniform distribution of aspirations between the two payoffs, or, in other words, it behaves randomly.

When aspirations are initially higher than $1+\delta /2$, agents do not create correlations, and their coordination ratio is about $50\%$, which makes them end in an aspiration of $A_{i}\simeq 1+\delta /2$. All their behavior is randomized, independently of the value of *h*. The only behavior that they share with the previous scenario is the non-Markovian one. In addition, the results for fragmented populations are similar to this. As aspirations are now dynamic, the important fact now is the amount of agents that can create correlations, as this is the behavior that can promote high coordination ratios and high aspirations for the agents.

To sum up, habituation moves aspirations towards the payoff average. This payoff average is $\ge 1+\delta /2$, where correlations can not be created. The specific evolution of the system depends on initial conditions.

### Small groups

As another feature to complete our study of this model, it is important to consider smaller group sizes for two reasons: First, if the model is to apply to early stages of human history, such as hunter-gatherer groups, the typical number of individuals involved in the process may certainly be smaller than the one considered so far. Second, it is possible to check the model predictions and assess how applicable the model is to actual situations by using experimental studies of our setup, and small numbers are easier to handle in that case. After carrying out simulations for smaller groups (number of agents = $\left\{ 200, 500, 1000, 1250, 1500 \right\}$), qualitative results do not change. This includes the effects arising from the variations on the learning rate, biasing, habituation and fragmented aspirations are the same, as they do not affect the stimuli structure.

However, the smaller size may give rise to smoothening of the transitions between regimes that we observed in Fig. 4, and one could in fact ask if there is some kind of Ising-like transition^34^ between the different regimes we have described. Figure 13 presents a plot of the region around $A_{i}=1+\delta /2$ for several sizes of the system. We will focus on the learning-random walker region ($A_{i}\simeq 1+\delta /2$). The other important region ($A_{i}=1$) has an easier analysis. The peak in the variance tends to be smaller as we increase the size, giving signals of a finite size effect. There are more reasons, related with the variation of the learning rate and the behavior of frequentists. The region ($A_{i}\simeq 1+\delta /2$) promotes a qualitative change of behavior, which we could not relate to any parameter of the system. However, as we can see in the picture below, the system does not exhibit the typical finite size scaling one would expect if we were facing a face transition.
